# Performance of a Mobile Single-Lead Electrocardiogram Technology for Atrial Fibrillation Screening in a Semirural African Population: Insights From “The Heart of Ethiopia: Focus on Atrial Fibrillation” (TEFF-AF) Study

**DOI:** 10.2196/24470

**Published:** 2021-05-19

**Authors:** Bradley M Pitman, Sok-Hui Chew, Christopher X Wong, Amenah Jaghoori, Shinsuke Iwai, Gijo Thomas, Andrew Chew, Prashanthan Sanders, Dennis H Lau

**Affiliations:** 1 Centre for Heart Rhythm Disorders The University of Adelaide Adelaide Australia; 2 Flinders University Adelaide Australia

**Keywords:** atrial fibrillation, screening, sub-Saharan Africa, single-lead ECG

## Abstract

**Background:**

Atrial fibrillation (AF) screening using mobile single-lead electrocardiogram (ECG) devices has demonstrated variable sensitivity and specificity. However, limited data exists on the use of such devices in low-resource countries.

**Objective:**

The goal of the research was to evaluate the utility of the KardiaMobile device’s (AliveCor Inc) automated algorithm for AF screening in a semirural Ethiopian population.

**Methods:**

Analysis was performed on 30-second single-lead ECG tracings obtained using the KardiaMobile device from 1500 TEFF-AF (The Heart of Ethiopia: Focus on Atrial Fibrillation) study participants. We evaluated the performance of the KardiaMobile automated algorithm against cardiologists’ interpretations of 30-second single-lead ECG for AF screening.

**Results:**

A total of 1709 single-lead ECG tracings (including repeat tracing on 209 occasions) were analyzed from 1500 Ethiopians (63.53% [953/1500] male, mean age 35 [SD 13] years) who presented for AF screening. Initial successful rhythm decision (normal or possible AF) with one single-lead ECG tracing was lower with the KardiaMobile automated algorithm versus manual verification by cardiologists (1176/1500, 78.40%, vs 1455/1500, 97.00%; *P*<.001). Repeat single-lead ECG tracings in 209 individuals improved overall rhythm decision, but the KardiaMobile automated algorithm remained inferior (1301/1500, 86.73%, vs 1479/1500, 98.60%; *P*<.001). The key reasons underlying unsuccessful KardiaMobile automated rhythm determination include poor quality/noisy tracings (214/408, 52.45%), frequent ectopy (22/408, 5.39%), and tachycardia (>100 bpm; 167/408, 40.93%). The sensitivity and specificity of rhythm decision using KardiaMobile automated algorithm were 80.27% (1168/1455) and 82.22% (37/45), respectively.

**Conclusions:**

The performance of the KardiaMobile automated algorithm was suboptimal when used for AF screening. However, the KardiaMobile single-lead ECG device remains an excellent AF screening tool with appropriate clinician input and repeat tracing.

**Trial Registration:**

Australian New Zealand Clinical Trials Registry ACTRN12619001107112; https://www.anzctr.org.au/Trial/Registration/TrialReview.aspx?id=378057&isReview=true

## Introduction

Consumer use of wearable technology capable of ambulatory assessment of heart rate and rhythm has significantly increased in recent years [1]. Large-scale population screening studies have demonstrated the capability of wearable devices to detect pulse irregularity using photoplethysmography-based technology, with a high positive predictive value of diagnosing atrial fibrillation (AF) [2,3]. However, the adoption of these smart wearable devices is much lower in low-resource countries due to affordability and low internet penetration rate. Despite AF being recognized as a growing global epidemic, the 2010 Global Burden of Disease study has highlighted low availability of data on AF from several regions including sub-Saharan Africa and the need for better estimates through targeted population surveillance studies [4]. Alternative active screening strategies for AF using pulse palpation and electrocardiogram (ECG) are therefore more applicable in these low-resource countries [1,5].

AF screening using single-lead ECG devices has been reported in hospital, primary care, and community settings with variable sensitivity and specificity [6]. However, limited data exist on the use of such devices for AF screening in low-resource countries [7]. One such device is the KardiaMobile ECG monitor (AliveCor Inc), which is approved by the US Food and Drug Administration (FDA) for automatic classification of 30-second single-lead ECG tracing as normal or possible AF. However, the device also returns other results of too short, tachycardia, bradycardia, unreadable, or unclassified. Notably, screening studies using the KardiaMobile device, including the Heart Rhythm Society/American College of Physicians AF Screening and Education Initiative, have encountered between 5% and 28% of unclassified ECG recordings [8-12]. The high frequency of unclassified tracings may limit the effective utility of this device for AF screening. Here, we sought to determine the real-world feasibility and utility of the KardiaMobile single-lead ECG device for AF screening in a semirural African population. Specifically, this analysis evaluates the device’s accuracy for AF detection, factors underlying unclassified ECG tracings, and factors that may influence its screening performance from the first 1500 subjects recruited in the ongoing TEFF-AF (The Heart of Ethiopia: Focus on Atrial Fibrillation study).

## Methods

### TEFF-AF Study

**Figure 1:**
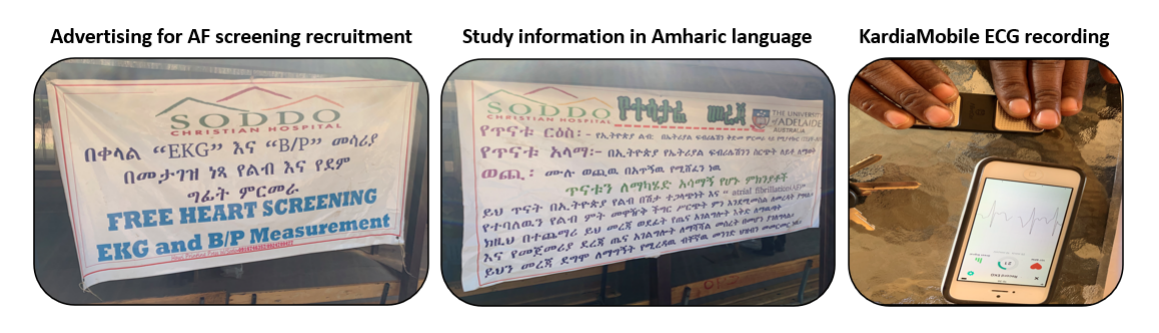
Atrial fibrillation screening advertising (left) and study information (center, in Amharic language) and single-lead electrocardiogram recording (right).

The TEFF-AF study (registered with the Australian New Zealand Clinical Trials Registry [ACTRN12619001107112]) is an AF screening study conducted at the Soddo Christian Hospital (SCH). The SCH is located in the semirural town of Soddo in south-central Ethiopia, with a population of around 200,000 individuals. AF screening was undertaken by a team of 5 nursing and research support staff from the SCH following specialized training on the use of the KardiaMobile device, iPhone app (version 5.7.4, KardiaAI: 1.1.7), and online Research Electronic Data Capture database. The training included an initial tutoring session followed by subsequent hands-on practice in acquiring a best-quality single-lead ECG tracing with the KardiaMobile device. AF screening commenced at the SCH in August 2019 with inclusion criteria being any ambulant adult aged 18 years and above and able to provide informed consent. Signage in Amharic language was erected to advertise screening to aid recruitment (Figure 1).

**Figure 2:**
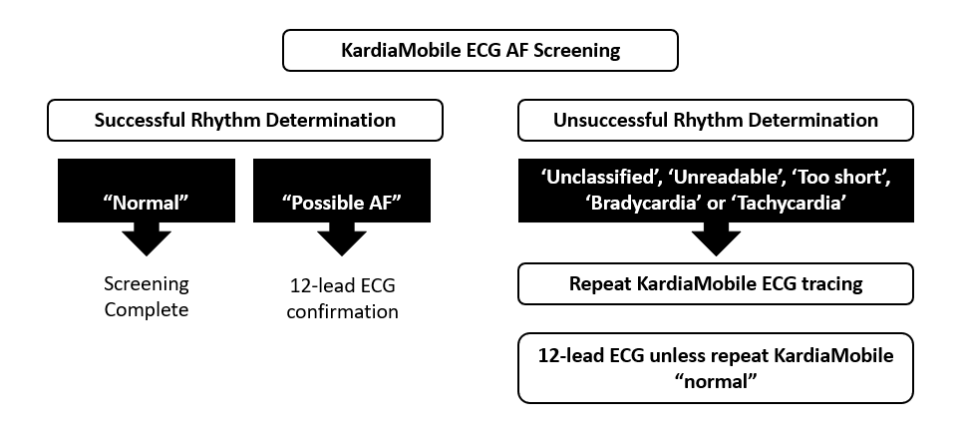
Atrial fibrillation (AF) screening protocol. ECG: electrocardiogram.

All participants provided informed consent, and the study is approved by the SCH research ethics board. Baseline demographic and clinical parameters were obtained to characterize the cardiovascular risk profile of participating individuals. Measurements of height, weight, and blood pressure (Intellisense T5 automatic monitor, Omron Corporation) were obtained before single-lead ECG acquisition using the KardiaMobile device. As per the study protocol (Figure 2), the outcome of the automated algorithm assessment of rhythm dictated the need for repeat KardiaMobile tracing and/or a 12-lead ECG. Participants with clinical abnormality detected were referred for follow-up by the SCH physician.

### KardiaMobile ECG Screening

The KardiaMobile mobile single-lead ECG device records a bipolar lead I ECG tracing when 2 or 3 fingers from each hand of the user are placed in contact with the 2 electrodes (Figure 1). Participants were instructed to relax arms and hands to reduce noise and artefacts. The KardiaMobile device transmits a frequency modulated ultrasound signal that is detected by the smartphone (iPhone, Apple Inc) with installed Kardia app. A 30-second single-lead ECG recording can be viewed in real time on the smartphone app and saved as a PDF file. The noise-filtered trace and computer-averaged complex on the KardiaMobile app is then subjected to an automated algorithm for arrhythmia diagnosis using the 2 criteria of p-wave absence and R-R interval irregularity [13].

### ECG Adjudication Analysis

The KardiaMobile ECG tracings obtained for the first consecutive 1500 participants in the TEFF-AF study were included in this analysis. Each single-lead ECG tracing has a rhythm determination by the KardiaMobile automated algorithm of normal, possible AF, bradycardia, tachycardia, unclassified, unreadable, or too short. Single-lead ECG traces were downloaded and analyzed independently by two cardiologists. The cardiologists also assessed diagnostic limitations for each tracing categorized as artefact, ectopy, bradycardia, tachycardia, or insufficient sample duration.

### Data Availability

The dataset with deidentified information generated and analyzed during this study is available from the corresponding author on reasonable request.

### Statistical Analysis

Summary statistics were presented by frequency and percentage or mean and standard deviation as appropriate. Categorical data were analyzed using the chi-square test. Sensitivity and specificity for the ability of the KardiaMobile to produce a rhythm decision against the cardiologist ECG interpretation was calculated. Linear regression analysis was performed to assess the factors contributing to screening performance of the KardiaMobile automated algorithm. All statistics were performed in SPSS Statistics version 26 (IBM Corp), and statistical significance set at *P*<.05.

## Results

### Participants

A total of 1709 single-lead ECG tracings (including repeat tracing on 209 occasions) were analyzed from a cohort of 1500 participants who presented for AF screening. The baseline clinical parameters of the participants are shown in Table 1. The mean age was 35 (SD 13) years and 63.53% (953/1500) were male. Of these participants, 95.93% (1439/1500) were from the regional state of Southern Nations, Nationalities, and Peoples’ Region where the SCH is located, and 87.07% (1306/1500) had secondary level education or above. The self-reported clinical history of the participants is shown in Table 1, with hypertension (104/1500, 6.93%) as the most prevalent comorbidity.

**Table 1:** Baseline clinical characteristics (n=1500).

Demographic and clinical information			Values
Age in years, mean (SD)			35 (13)
Gender, male, n (%)			960 (64.00)
**Home region, n (%)**			
	Southern Nations, Nationalities, and Peoples’ Region	1439 (95.93)	
	Omoria	30 (2.00)	
	Amhara	11 (0.73)	
	Other regions (including Somalia, B-Gumuz, Addis Ababa, Harar)	19 (1.27)	
**Religion, n (%)**			
	Orthodox	416 (27.73)	
	Protestant	988 (65.87)	
	Muslim	70 (4.67)	
	Other or no religion	22 (1.47)	
**Education, n (%)**			
	Illiterate	55 (3.67)	
	Primary level school	137 (9.13)	
	Secondary level school	599 (39.93)	
	Certificate, diploma, or higher	707 (47.13)	
**Occupation, n (%)**			
	Unemployed	175 (11.67)	
	Employed	682 (45.47)	
	Self-employed	344 (22.93)	
	Others including student and retired	297 (19.80)	
**Clinical, mean (SD)**			
	Height (cm)	167.7 (8.6)	
	Weight (kg)	67.1 (13.3)	
	Systolic blood pressure (mm Hg)	124.0 (17.7)	
	Diastolic blood pressure (mm Hg)	76.5 (11.7)	
**Clinical, n (%)**			
	Hypertension	104 (6.93)	
	Diabetes mellitus	34 (2.27)	
	Congestive cardiac failure	20 (1.33)	
	Stroke	3 (0.20)	
	Coronary artery disease	2 (0.13)	
	Peripheral artery disease	0 (0.00)	
	Chronic lung disease	16 (1.07)	
	Chronic renal disease	5 (0.33)	
	Valvular heart disease	11 (0.73)	
	Obstructive sleep apnea	2 (0.13)	
	Thyroid disease	21 (1.40)	
	Smoker	5 (0.33)	
	Khat/alcohol use	14 (0.93)	
	Infectious disease	288 (19.20)	

### Performance of the KardiaMobile Automated Algorithm

**Figure 3:**
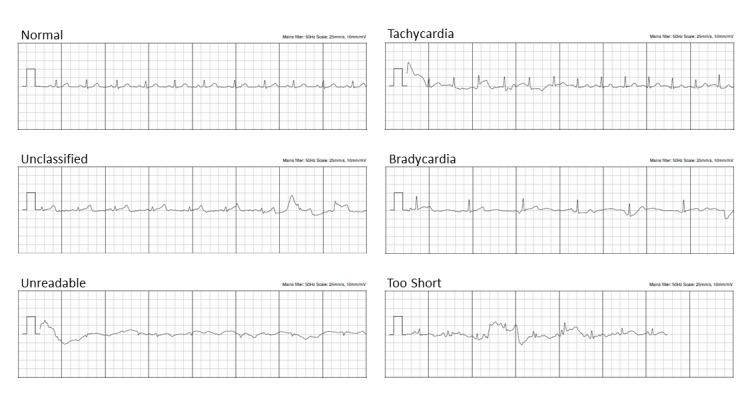
Examples of KardiaMobile single-lead electrocardiogram tracings.

Of the initial single-lead ECG tracings from 1500 participants, the KardiaMobile algorithm was unable to provide a rhythm decision in 21.60% (324/1500) due to unclassified (130/1500, 8.67%), tachycardia (128/1500, 8.53%), unreadable (62/1500, 4.13%), too short (3/1500, 0.20%), and bradycardia (1/1500, 0.07%). Representative examples of these tracings are shown in Figure 3. A repeat KardiaMobile tracing was obtained in 64.50% (209/324) of the participants who did not have an initial rhythm decision. Of those participants without repeat KardiaMobile tracings, 83.48% (96/115) had an initial result of tachycardia, which the screening team deemed as sinus tachycardia (>100 bpm) and interpreted as normal rhythm not requiring a repeat tracing, 10.43% (12/115) proceeded directly to a 12-lead ECG, and 6.09% (7/115) declined repeat KardiaMobile tracing or 12-lead ECG due to time constraint. On the repeat KardiaMobile attempt, the KardiaMobile algorithm again failed to achieve a rhythm decision in 40.19% (84/209). Adjudications by cardiologists showed that the reasons underlying unsuccessful automated KardiaMobile rhythm determination (n=408 traces; 324 from first attempt and 84 from repeat attempt) were poor quality/noisy tracings (214/408, 52.45%), tachycardia (>100 bpm; 167/408, 40.93%), frequent ectopy (22/408, 5.39%), inadequate recording duration (3/408, 0.74%), and bradycardia (<50 bpm; 2/408, 0.49%).

### KardiaMobile Automated Algorithm Versus Cardiologist Adjudication

**Figure 4:**
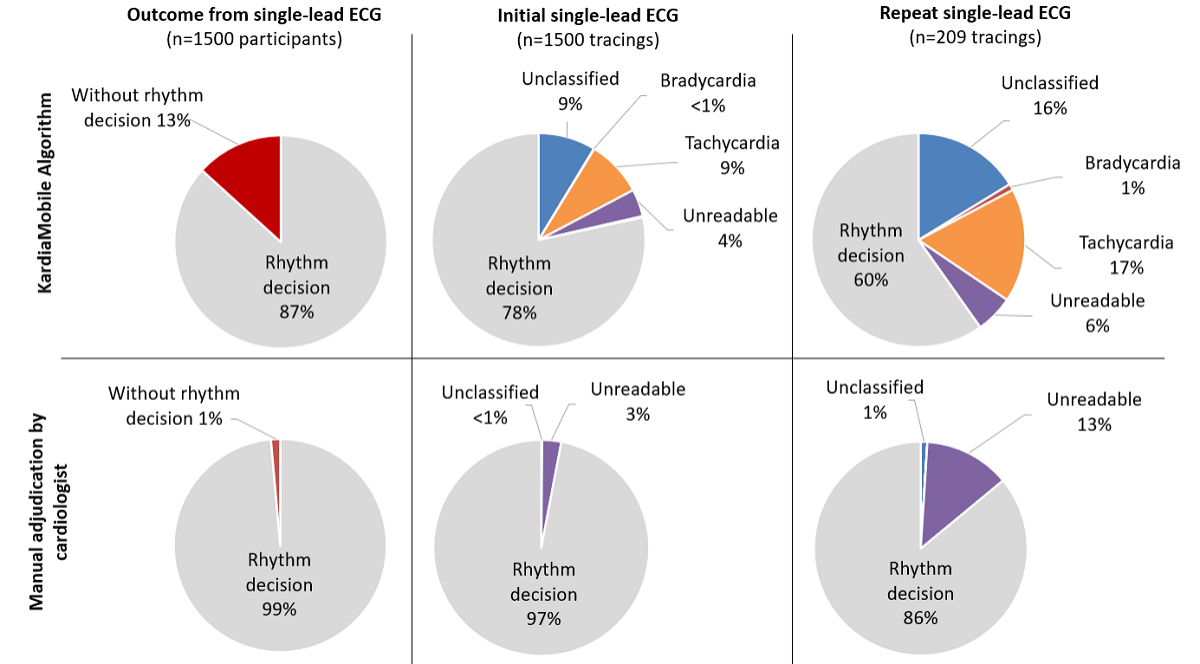
Comparison of KardiaMobile algorithm versus manual assessment by cardiologists. ECG: electorcardiogram.

The KardiaMobile automated algorithm successfully obtained a rhythm decision on the first attempt for 78.40% (1176/1500) of participants, which was considerably lower than manual assessment by cardiologists (1455/1500, 97.00%; *P*<.001; Figure 4). The sensitivity and specificity of a rhythm decision by the KardiaMobile automated algorithm from the initial single-lead ECG of each participant, when compared with manual assessment by cardiologists, was 80.3% (95% CI 78.1% to 82.3%) and 82.2% (95% CI 68.0% to 92.0%), respectively (Table 2). The KardiaMobile automated algorithm’s success in rhythm decision improved to 86.73% (1301/1500) with the inclusion of repeat KardiaMobile tracings achieving a rhythm decision for an additional 125 participants, although it remained lower than manual assessment by cardiologists (1479/1500, 98.60%; *P*<.001; Figure 4). In total, 96.96% (1657/1709) of the single-lead ECG tracings were of adequate quality for diagnostic purposes according to cardiologist adjudication. Notably, all the KardiaMobile algorithm-determined normal single-lead ECG tracings were confirmed to be normal sinus rhythm according to cardiologist adjudication. However, 3 traces that failed to achieve a rhythm decision by KardiaMobile (2 unreadable and 1 unclassified) were deemed AF by cardiologist adjudication. The sensitivity and specificity of AF detection by the KardiaMobile automated algorithm from 1709 single-lead ECG tracings, when compared with manual assessment by cardiologists, was 75.0% (95% CI 42.8% to 94.5%) and 96.4% (95% CI 95.4% to 97.2%), respectively (Table 2).

**Table 2:** KardiaMobile automated algorithm versus cardiologists’ adjudication for single-lead electrocardiogram (ECG) for rhythm decision in n=1500 participants and atrial fibrillation detection in n=1709 ECG tracings.

KardiaMobile algorithm			Cardiologists’ adjudication						
			Rhythm decision				Atrial fibrillation		
			Yes		No		Yes		No
**Rhythm decision^a^**									
	Yes	1168		8		—^b^		—	
	No	287		37		—		—	
**Possible atrial fibrillation^c^**									
	Yes	—		—		9		61	
	No	—		—		3		1636	

^a^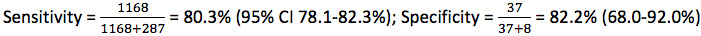.

^b^Not applicable.

^c^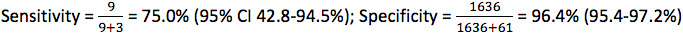.

### 12-Lead ECG Analysis

In total, 154 participants met criteria for a 12-lead ECG, but this was obtained in only 59.09% (91/154) due to participants not wanting to wait for the 12-lead ECG to be performed in the SCH emergency room. However, upon review of the single-lead ECGs meeting study criteria for a 12-lead ECG to be performed, the cardiologists adjudicated 89.61% (138/154) of these single lead ECGs to be of adequate quality for a rhythm decision. In total, diagnoses from the 12-lead ECGs were 89.01% (81/91) sinus rhythm, 1.10% (1/91) supraventricular tachycardia, and 9.89% (9/91) AF.

### Utility of KardiaMobile Automated Algorithm for AF Screening

**Figure 5:**
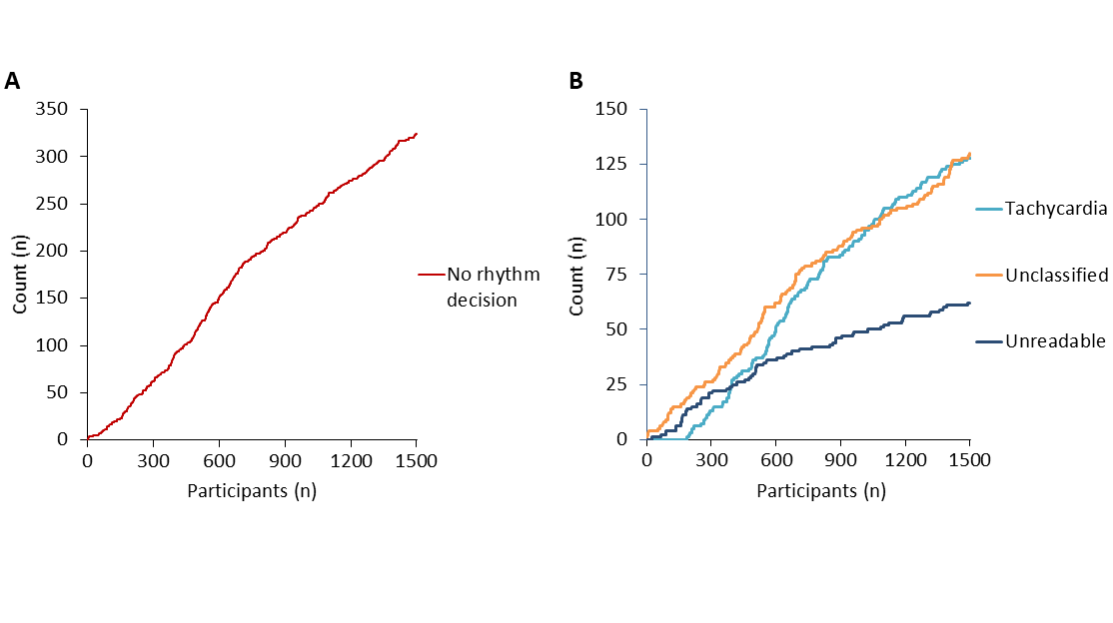
Cumulative occurrence and contributors to no rhythm decision from KardiaMobile’s automated algorithm on initial electrocardiogram tracing: (A) cumulative occurrence of no rhythm decision from initial electrocardiogram tracing and (B) occurrence of unreadable tracing was significantly reduced when compared with unclassified and tachycardia tracings with increasing patient recruitment.

We analyzed the performance of the KardiaMobile automated algorithm for providing an initial rhythm decision. There was a linear relationship between ongoing participant recruitment and the occurrence of a no rhythm decision from the initial KardiaMobile tracing (Figure 5A). Linear regression analysis showed that there was a significant reduction in the cumulative incidence of no rhythm decision compared with successful rhythm decision with ongoing patient recruitment (*β*=–14.4, 95% CI –26.6 to –2.1; *P*=.02). As the KardiaMobile results of tachycardia, unclassified, and unreadable accounted for 98.77% (320/324) of occasions without a rhythm decision on the first KardiaMobile attempt, their contribution to no rhythm decision was further analyzed. With ongoing patient recruitment, the occurrence of unreadable tracing was significantly reduced when compared with unclassified and tachycardia tracings (*β*=–38.0, 95% CI –63.3 to –12.6; *P*=.003, Figure 5B).

## Discussion

### Principal Findings

This study evaluated the utility of the KardiaMobile single-lead ECG device for AF screening in a semirural Ethiopian population of 1500 individuals from the TEFF-AF study. We found the KardiaMobile device performance to be suboptimal with successful automated rhythm decision following a single ECG trace of only 78%. This yield increased to 87% following a second KardiaMobile ECG tracing. As experience increased with ongoing patient recruitment, we encountered significant reduction in unreadable tracings. The ongoing occurrence of tachycardia and unclassified tracings contributed largely to the automated KardiaMobile algorithm’s inability to achieve successful rhythm decision. In contrast, manual cardiologist assessment was able to obtain a rhythm decision in almost all cases (97%) with a single ECG. Taken together, our findings suggest that manual physician input remains necessary when the KardiaMobile device is used for AF screening.

The use of single-lead ECG devices is of increasing interest given the potential benefits of portability and scalability. Furthermore, automated rhythm analysis may allow for the use of such devices by individuals without formal medical training. However, there are limited data on the accuracy of these devices and their automated rhythm analysis algorithms in such settings despite the KardiaMobile device having been FDA-approved since 2012. In a small validation study, the KardiaMobile’s automated AF detection algorithm was reported to yield high sensitivity of 98% and specificity of 97% with overall accuracy of 97% [13]. In a single-center, adjudicator-blinded case series of 52 consecutive patients with AF admitted for antiarrhythmic drug initiation who had serial 12-lead ECG and nearly simultaneously acquired KardiaMobile recordings, AF detection was reported at 96.6% sensitivity and 94.1% specificity [14]. However, 28% of the tracings obtained were deemed unclassified by the KardiaMobile automated algorithm and excluded from analysis. Similarly, others have reported the KardiaMobile automated algorithm correctly detected AF with 93% sensitivity and 84% specificity in 100 participants with a history of AF who presented for a scheduled elective electrical cardioversion after excluding a substantial 34% of recordings with unclassified tracings [11]. Our study found that the KardiaMobile automated algorithm failed to achieve rhythm decision in 22% of the tracings, comparable to previous studies. Consequently, this may limit the utility of the mobile single-lead ECG device for mass AF screening and opportunity to offer oral anticoagulation for stroke prevention in those with newly detected AF. It remains unclear if another mobile single-lead ECG device that was found to have higher sensitivity and similar specificity when compared with the KardiaMobile device will yield better AF screening performance [15].

Recently, several studies have reported on the use of other smart wearable devices using photoplethysmography-based technology for AF screening. The Apple Heart Study reported on the ability of a smartwatch photoplethysmography sensor and algorithm to screen individuals for an irregular pulse. Of 419,297 individuals, 2161 (0.52%) had a smartwatch-detected irregular pulse, with AF confirmed in 34% of those who returned an ECG patch. Of the individuals who had a smartwatch-detected irregular pulse while simultaneously wearing an ECG patch, 84% (78/86) were in AF at the time [2]. The Huawei Heart Study similarly described the use of smartwatch or smartband photoplethysmography to screen 187,912 individuals. Of 227 with suspected AF who underwent complete history, examination, and ECG or 24-hour Holter monitoring, 87% were confirmed to have AF [3]. Although these data highlight the utility of automated algorithms to flag possible AF, both studies still incorporated physician review of confirmatory traces, and there remains a paucity of data comparing photoplethysmography-based and single-lead ECG technology.

### Clinical Implications

Our study has important clinical implications for AF screening and highlights opportunities for future research. Prior research has shown that automated device algorithms can achieve accurate rhythm analysis under ideal conditions. However, our real-world experience in a resource-limited setting demonstrates that single-lead ECG tracing artefact and other limiting factors frequently prohibits algorithm interpretation. Despite limitations with tracing quality, manual cardiologist adjudication can still provide a rhythm diagnosis in the vast majority of cases. Thus, our findings suggest that physician input remains necessary for AF screening until further improvements in automated algorithms occur. In the meantime, repeat ECG tracings and increasing familiarity with using single-lead ECG devices are helpful to reduce unreadable tracings to improve diagnostic yield. Future studies should be undertaken to validate other mobile device technology and automated algorithms in real-world settings.

### Limitations

Our screening protocol required a repeat tracing for occasions without a rhythm decision. However, this was not performed in a proportion of the participants, resulting in an incomplete data set. We acknowledge that the clinical value of AF screening in a young cohort with unknown risk factors for stroke is unclear. Nevertheless, given the knowns and unknowns of AF in sub-Saharan Africa and the higher prevalence of rheumatic heart disease, we did not restrict the AF screening to the typical target population of older individuals with higher stroke risk in developed countries [16]. As with all single time point AF screening, paroxysmal AF may be missed, leading to false negatives. Although our liberal inclusion criteria did achieve a diverse sample of the local community, we acknowledge that our data may not reflect the true prevalence of AF in this community due to the recruitment site being based at a local hospital.

### Conclusion

The performance of the automated algorithm of the KardiaMobile single-lead ECG device was suboptimal when used for AF screening. However, the KardiaMobile device remains an excellent and affordable tool when used in low-resource settings with appropriate clinician input.

## Abbreviations

AF: atrial fibrillation
ECG: electrocardiogram
FDA: US Food and Drug Administration
KM: KardiaMobile
SCH: Soddo Christian Hospital
TEFF-AF: The Heart of Ethiopia: Focus on Atrial Fibrillation study
